# Pentacyclic Triterpenoid Content in Cranberry Raw Materials and Products

**DOI:** 10.3390/foods13193136

**Published:** 2024-09-30

**Authors:** Liang Xue, Bianca Carreiro, Md Sagir Mia, Inke Paetau-Robinson, Christina Khoo, Catherine Neto

**Affiliations:** 1 Department of Chemistry and Biochemistry and Cranberry Health Research Center, University of Massachusetts Dartmouth, North Dartmouth, MA 02747, USA; liangxue2014@gmail.com (L.X.); bcarreiro2@umassd.edu (B.C.); mmia@umassd.edu (M.S.M.); 2 Ocean Spray Cranberries, Inc., Lakeville, MA 02349, USA; irobinson@companapetbrands.com (I.P.-R.); ckhoo@oceanspray.com (C.K.)

**Keywords:** cranberry pomace, ursolic acid, oleanolic acid, triterpenoids, cranberry juice, juice processing

## Abstract

Cranberry fruit extracts have been shown to inhibit expression of pro-inflammatory cytokines in THP-1 cells and reduce colonic tumor burden and tissue inflammation in a mouse model of colitis. These activities are attributed to both the triterpenoid and polyphenol constituents of the fruit. The pentacyclic triterpenoids ursolic acid (UA), oleanolic acid (OA), corosolic acid (CA), maslinic acid (MA), and esters of UA and OA occur in the waxy layer of cranberry peel, and their content in cranberry products is likely to vary with the fruit source and processing methods. UPLC-MS (ultra performance liquid chromatography-mass spectrometry) was applied to determine the four triterpenoid acids and their esters in cranberry products and raw materials. Cranberry pomace, a side stream in juice production, was a rich source at 64,090 µg total triterpenoids/g DW. Cranberry juice beverages ranged from 0.018 to 0.26 µg/g of product, fruit samples ranged from 6542 to 17,070 µg/g DW, and whole berry products contained up to 2665 µg/g DW. Free UA was the most plentiful triterpenoid in all samples. These analyses illustrate the potential value of an underutilized side stream in cranberry juice production and highlight potential benefits of whole fruit products.

## 1. Introduction

**Figure 1:**
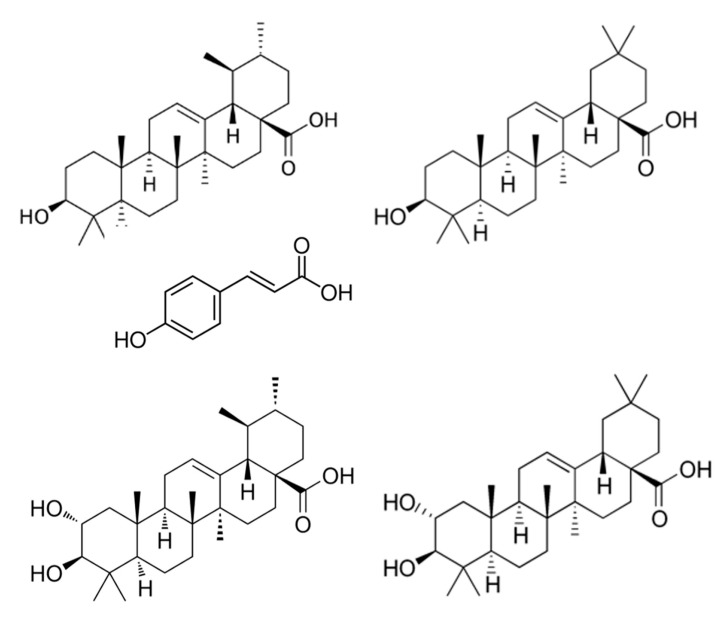
Structures of ursolic acid (UA, **upper left**), oleanolic acid (OA, **upper right**), p-hydroxycinnamic acid (**middle**), corosolic acid (CA, **lower left**), and maslinic acid (MA, **lower right**).

Cranberries contain a variety of pentacyclic triterpenoids with potential health benefits, principally ursolic acid (UA) and oleanolic acid (OA). UA, reported to possess a variety of biological effects including antiproliferative [1], anti-inflammatory [2], and neuroprotective antioxidant properties [3], is found in a number of edible fruits and herbs, such as apple and thyme, as well as many medicinal plants [4]. UA, OA, and their derivatives hold promise for improving cardiovascular health [4], cancers, and skin conditions [5]; thus, there is interest in identifying natural sources. UA is present in both free and esterified forms in cranberry fruit [6]. The p-hydroxycinnamoyl esters of UA are of particular interest, as we previously reported their ability to inhibit cell proliferation in multiple tumor cell lines and inhibit expression of MMP-2 and MMP-9 in prostate tumor cells [6]; increased expression of MMPs is associated with the invasive and metastatic nature of prostate cancer [7]. Cranberry also contains the 2-hydroxy derivatives of UA and OA, corosolic acid (CA), and maslinic acid (MA) (Figure 1), compounds which may contribute to its antiproliferative and antioxidant activities [8,9]. We have previously reported that extracts of cranberry fruit rich in these four triterpenoid acids inhibit expression of pro-inflammatory cytokines in THP-1 monocytes, and thus these compounds may play a role in the observed ability of cranberry extract to limit inflammatory processes [10]. UA and OA are major constituents of an ethyl acetate-soluble extract of cranberry fruit, which we found significantly reduced colonic tumor burden and tissue inflammation in a mouse model of colitis [11]. As these triterpenoids are components of the waxy layer of cranberry peel, their content in cranberry products is likely to vary depending on the source and processing methods. For example, a recent study of phytochemicals in commercial cranberry supplements found wide variation in the content of UA and OA as well as other components plentiful in the peel [12]. It is therefore important to understand the content and distribution of these compounds in cranberry raw materials and products.

Previous studies have demonstrated that cranberry triterpenoids can be quantified effectively by UPLC-DAD (ultra performance liquid chromatography with diode-array detection) or UPLC-MS [6,13,14]. We recently applied UPLC-MS to determine the content of four triterpenoid acids in extracts of cranberry fruit that reduced inflammation in THP-1 monocytes [10]. The goal of the present study was to determine the content of the major biologically active triterpenoid acids and their hydroxycinnamoyl esters in cranberry fruit sourced in North America, in solid materials resulting from the processing of fruit to make beverages and sweetened dried fruit, and in a variety of commercial cranberry products that may serve as dietary sources of these compounds. Herein we report the application of UPLC-MS to determine four pentacyclic triterpenoid acids (UA, OA, CA, MA) and hydroxycinnamoyl esters of UA and OA in a range of cranberry products, raw materials, and fruit samples of different cultivars and demonstrate that cranberry fruit and raw materials from the juice processing side stream can be a plentiful source of these compounds.

## 2. Materials and Methods

### 2.1. Plant Materials and Reagents

Cranberry fruit of several cultivars (Stevens, Early Black, GH1, and Welker) was harvested in 2016 or 2017 from two locations in the United States: the UMass State Bog in East Wareham, Massachusetts, in late September (authenticated by Krystal DeMoranville, UMass Cranberry Station), and Bandon, Oregon, in late October (authenticated by Cassie Bouska, Oregon State University Extension Service). Cranberry samples were selected randomly within each cultivar plot. Fruits were flash frozen with liquid nitrogen and stored at −20 °C until use. Cranberry materials, including pomace (Lot 10182018), a by-product of the juicing process including the hulls, and presscake (Lot 03092020), a by-product of dried cranberry production including pieces of the fruit, were produced at Ocean Spray Cranberries, Inc. from fruit of mixed cultivars (Stevens, Early Black, and others) harvested in Massachusetts. Yellow Bell variety fruit was obtained by Ocean Spray from growers in Wisconsin. Commercial cranberry-derived products, including sweetened dried fruit (extracted and infused dried cranberries), vacuum-fried whole cranberries, whole berry cranberry sauce (containing whole berries in addition to jellied juice concentrate), and commercially available cranberry juice cocktail beverages, were provided by Ocean Spray Cranberries, Inc. All raw materials were stored at −20 °C until use; products were stored at 4 °C until use. Authentic standards of UA and OA were purchased from Sigma-Aldrich (St. Louis, MO, USA); MA and CA were purchased from Extrasynthese (Genay, France). Reagent grade ethanol, methanol, ethyl acetate and acetone, and UV/HPLC grade acetonitrile and methanol were purchased from Pharmco-AAPER (Brookfield, CT, USA). HPLC grade water was purchased from Honeywell (Morristown NJ, USA).

### 2.2. Sample Preparation

All fruit, solid materials, and products (including fried cranberry, presscake, pomace, sweetened dried fruit, and whole cranberry sauce) were freeze-dried with liquid nitrogen and ground to a powder using a mortar and pestle and a coffee grinder. Extract preparation was carried out in a similar manner to our previous studies on cranberry fruit metabolites [10,15], with some changes. Methanol was used as a suitable universal solvent for these analytes that is also compatible with the UPLC method, as most extracts were not dried or reconstituted prior to analysis. A total of 100 mg aliquots of each powdered sample was prepared by extraction with 10 mL of reagent grade methanol in a small tube at room temperature with sonication using an ultrasonicator (Fisher Scientific FS20H, Pittsburgh, PA, USA) for 20 min with periodic shaking. After centrifugation, the supernatants were collected, and the solid residue was re-extracted with 10 mL of methanol, sonicated, and centrifuged as described above. The extraction process was repeated a total of three times. Supernatants were combined and filtered through a 0.45 mm PTFE syringe filter and stored in a sealed tube at −20 °C prior to UPLC-MS analysis. Each sample was prepared and analyzed in triplicate, diluting to the appropriate concentration for detection within the standard curve concentration range for the major analytes as given in Section 2.3. In cases where the initial solution was too dilute to quantify all analytes of interest, samples were dried in vacuo and reconstituted with methanol to the appropriate concentration to detect the analytes. 

Based on previous studies, cranberry juices were expected to have lower triterpenoid content than other products; thus, samples were concentrated prior to analysis using previously published methods [6] by partitioning with ethyl acetate, removing water-soluble constituents, and concentrating the triterpenoids in the organic layer. Juice samples (200 g) were mixed vigorously with ethyl acetate (100 mL), stirred magnetically at room temperature for 10 min, then transferred into a separatory funnel. The ethyl acetate layer was washed twice with 50 mL of deionized water and collected. The water layer was re-extracted twice with ethyl acetate (100 mL). All organic layers were combined and rotary evaporated to dryness. Extracts were prepared and analyzed in triplicate and re-constituted with methanol at the appropriate concentration range for analysis.

### 2.3. UPLC-MS and Data Analysis

An UPLC-MS method was developed previously to determine the content of four triterpenoid acids associated with anti-inflammatory activity in cranberry extracts: UA, OA, MA, and CA [10]. For the present study, the method was modified to also detect hydroxycinnamoyl esters (cis- and trans-3-O-p-hydroxycinnamoyl UA) previously reported by us as bioactive constituents in cranberries [11]. Briefly, the method employed a Waters Xevo QTOF mass spectrometer (Waters Corporation, Milford, MA USA) with electrospray ionization source operating in negative ion mode, and an ACQUITY UPLC system, equipped with a Waters ACQUITY UPLC BEH C18 column (Waters Corporation, Milford, MA, USA) (1.7 μm, 2.1 × 100 mm), held at a temperature of 25 °C. Isocratic elution proceeded with a mobile phase of 85:15 solvent A (0.1% formic acid in methanol) and solvent B (0.1% aqueous formic acid) at a flow rate of 0.3 mL/min. The effluent was monitored by ESI-MS in negative ion mode, in the mass range of *m*/*z* 50−1000 using a capillary voltage of 2 kV, a sampling cone voltage of 160 V, an extraction cone voltage of 6 V, a source temperature of 100 °C, a desolvation temperature of 345 °C, a cone gas flow of 50 L/h, and a desolvation gas flow of 600 L/h [10]. UA and OA were monitored at [M-H]^−^ = 455.4; CA and MA were detected at [M-H]^−^ = 471.4. Cis- and trans-hydroxycinnamoyl esters of UA and OA were monitored at [M-H]^−^ = 601.4. Data analysis and quantification were performed in comparison to the commercial standards, using MassLynx software, Version 4.1. Samples were analyzed in triplicate. Linear standard curves were prepared and analyzed each day for each triterpenoid with a concentration range of approximately 0.1–2 µg/mL for UA, 0.0375–1.2 µg/mL for OA, 0.020625–0.33 µg/mL for MA, and 0.025–0.8 µg/mL for CA. Pomace, presscake, and fruits were analyzed at concentrations ranges between 75 and 6000 µg/mL, cranberry products at 7500–20,000 µg/mL, and cranberry juices at 25,000–50,000 µg/mL. Analyses were conducted in triplicate, and results are reported as the mean +/− the standard deviation.

## 3. Results

**Figure 2:**
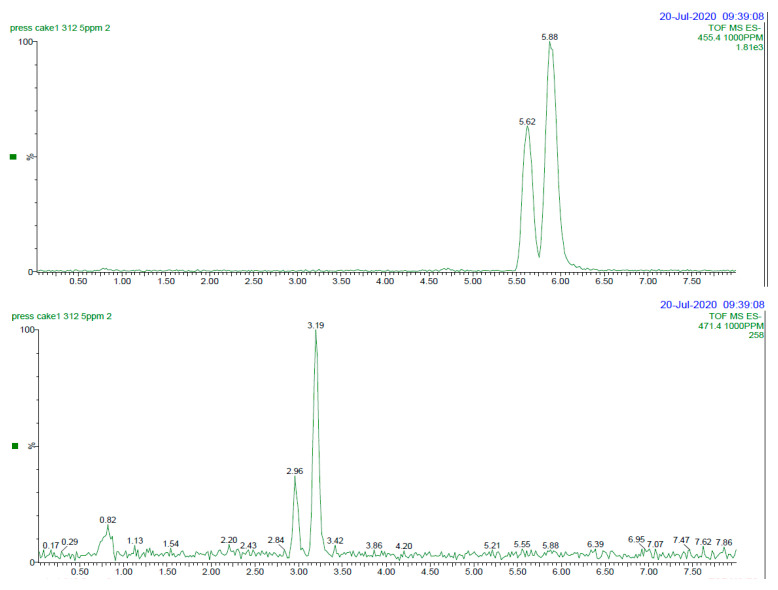
UPLC-MS extracted ion chromatograms. (**top**): cranberry presscake, monitored at [M-H]^−^ = *m*/*z* 455.4; peaks eluting at 5.62 and 5.88 min, monitored at [M-H]^−^ = *m*/*z* 455.4, were identified as oleanolic acid (OA) and ursolic acid (UA), respectively. (**bottom**): cranberry presscake, monitored at [M-H]^−^ = *m*/*z* 471; peaks eluting at 2.96 and 3.19 min were identified as maslinic acid (MA) and corosolic acid (CA), respectively. Mass spectra can be found in the Figures S1–S4.

The UPLC-MS method was applied to determine the content of four triterpenoid acids—UA, OA, CA, and MA (Figure 1)—as well as the hydroxycinnamoyl esters of UA and OA in cranberry raw materials and products. Figure 2 shows sample chromatograms for the four triterpenoid acids in cranberry presscake. The primary acids were identified by comparison to authentic commercial standards and quantified using standard curves acquired on the day of analysis. UA and OA, detected at [M-H]^−^ = 455.4, were typically eluted at 5.62 and 5.88 min, respectively, (Figure 2 top) using the UPLC-MS program described. Maslinic and corosolic acid, detected at [M-H]^−^ = 471.4, were typically eluted at 2.96 and 3.19 min, respectively (Figure 2, bottom).

**Figure 3:**
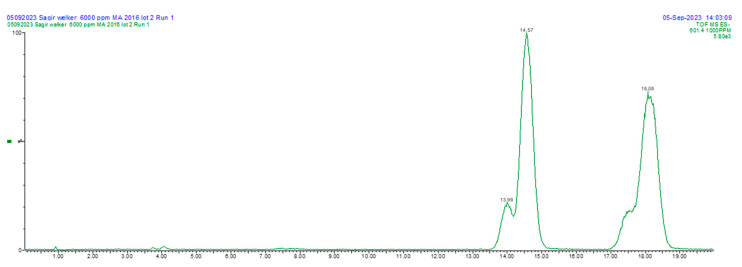
Massachusetts cranberry fruit, monitored at [M-H]^−^ = *m*/*z* 601.4; peaks shown were identified as cis- and trans-3-O-p-hydroxycinnamoyl esters of OA and UA. Mass spectra can be found in Figure S5 and Figure S6.

Peaks eluting between 13 and 19 min could be detected at [M-H]^−^ = 601.4 in most of the raw materials and fruit samples. The two major peaks were identified as cis- and trans-3-O-p-hydroxycinnamoyl UA (Figure 3), based on previous analyses [6,11]; their identities were previously verified by ^1^H NMR. Minor peaks eluting immediately prior are putatively identified as the cis -and trans-3-O-p-hydroxycinnamoyl esters of OA, and these analytes were present in a lesser quantity in most samples. As the peak resolution of the isomers was insufficient for some samples, we report the total content of the four esters. Table 1 summarizes the content of analytes in raw materials and products including the four juice samples on a wet weight basis, and Table 2 summarizes the content of analytes in fruit samples and other solid materials on a dry weight basis, expressed as micrograms per gram of product. All four triterpenoid acids were present in detectable and measurable quantities in these samples, with the exception of juice sample A. The cis-3-O-p-hydroxycinnamoyl and trans-3-O-p-hydroxycinnamoyl UA esters are also present in detectable quantities in all of the raw materials and products except for the four cranberry juice products.

Among all samples tested, the most plentiful source of triterpenoid acids was cranberry pomace, a by-product of the juicing process including the hulls, with 15,500 ± 1700 µg UA, 3990 ± 360 µg OA, 164 ± 4.9 µg MA, and 459 ± 21 µg CA per gram of pomace (wet weight), as seen in Table 1. Cranberry presscake, a by-product of dried cranberry production which includes pieces of the fruit, was also high in triterpenoids with 6494 ± 29 µg UA, 2133 ± 37 µg OA, 55.3 ± 3.3 µg MA and 207.5 ± 7.1 µg CA per gram wet weight. On a dry weight basis, the pomace sample contained over 64,000 µg of triterpenoid acids and esters per gram (Table 2), making up approximately 6.4% of the total pomace mass. Approximately 74.1% of the total triterpenoid in the cranberry pomace was free UA, with its isomer, OA, making up an additional 19.1%, and the combined hydroxycinnamoyl esters of UA and OA accounting for about 2.9%. 2-hydroxy derivatives of UA and OA corosolic and maslinic acid contributed 3% and 0.8%, respectively. The whole cranberry fruit samples analyzed varied widely in total triterpenoid content, between 6542 ± 119 µg per g DW for GH-1 cultivar from Oregon (2016 season) and 17,070 ± 320 µg per g DW for Early Black cultivar fruit harvested in Massachusetts (2017 season). The commercial cranberry products tested also varied widely; beverages varied somewhat but in general provided much lower triterpenoid levels than products containing whole fruit, such as whole berry sauce.

## 4. Discussion

Cranberry pomace is a side stream or by-product in the production of cranberry juice and is used as a fiber source for feed or pet food. Our results suggest the potential value of this underutilized side stream as a rich source of bioactive triterpenoids. Among the commercial products we tested, sweetened dried fruit (Craisins™) was a plentiful source of these compounds, providing about 2500 µg per gram or 100 mg triterpenoid per 40 g serving. Levels of UA and OA (Table 1) were higher than those reported by Zhang and coworkers in dried cranberries from Japan (660 µg of UA and 178 µg of OA per g of product) [16], which could be attributed to geographical differences among source fruit, as discussed below. Triterpenoid content in cranberry juice beverages tested ranged from 0.018 to 0.260 µg per gram of juice. The content in juice is likely limited by the low water solubility of these compounds. The range suggests that processing methods employed in producing juice and juice-derived products can influence triterpenoid content. UA was the most plentiful of the four acids in all juice samples at 13–191 ng/g juice, while MA was present in the lowest quantity with concentrations of between 0.7 and 7 ng/g in three of the juices tested. As these concentrations are below the limit for direct quantification by our instrument, the triterpenoids were extracted from beverages and concentrated for analysis using ethyl acetate. This yielded extracts with triterpenoid concentrations of between 0.5 and 33 µg per gram. However, the CA and MA content was still below the limit of detection in the extract for cranberry juice beverage A, and no UA esters could be detected in any of the juice samples tested. As cranberry juice is acidic, it is possible that the conditions employed in processing juices (e.g., heating for sterilization) could hydrolyze any ursolic esters present in the juice to their parent triterpenoid. 

**Figure 4:**
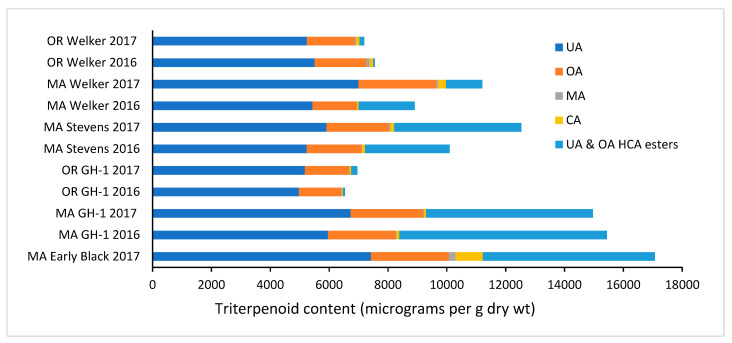
Comparison of triterpenoid distribution among cranberry fruit samples from two major North American growing regions and four cultivars.

In general, the content of hydroxycinnamoyl UA esters in raw materials and fruit samples varied widely. The majority of the UA in most samples is present in the free acid form, consistent with our previous studies [6]. The percentage of total UA found in the form of hydroxycinnamoyl esters was higher in cranberry presscake (11.6%) and many of the fruit samples than the percentage found in products such as whole berry cranberry sauce (2.0%) and sweetened dried fruit (4.4%), again suggesting that processing methods may affect the content of esters relative to the free triterpene acid; for example, the use of heat in the preparation along with the natural acidity could accelerate hydrolysis. The overall triterpenoid content of cranberry sauces would also depend on the amount of fruit peel retained in the product; a previous study reported much higher ursolic acid content in whole berry sauce than in jellied cranberry sauce [6]. Cranberry presscake and pomace raw materials were derived from the fruit of multiple cultivars harvested from East Coast bogs in 2018. We also compared selected cranberry fruit samples of five different cultivars and found wide variation in total triterpenoid content, distribution (Figure 4), and percentage of UA in esterified form. Across all samples, results confirm that UA is the major pentacyclic triterpenoid present in cranberry fruit, raw materials, and products, followed by OA. The content of MA and CA is generally much lower than UA or OA in all samples. The percentage of UA found as hydroxycinnamoyl esters varied widely among the fruit samples. Figure 4 compares the distribution of triterpenoids in eleven fruit samples, seven harvested in Massachusetts and four harvested in Oregon. Early Black cultivar fruit, a native selection from MA, had the highest overall triterpenoid content among the fruit tested. When comparing the samples available from both regions (GH-1 and Welker), the average total triterpenoid content was significantly higher for the fruit harvested in MA than that from OR. Much of the ursolic and OA detected in the MA fruit occurs in the form of hydroxycinnamoyl esters (1235–7055 µg/g), whereas the OR fruit contained much lower levels of esterified triterpenoids (58–211 µg/g).

The factors that determine the distribution of triterpenoids in cranberry fruits of various genetic origins are unknown at this time. A study of six cranberry cultivars grown in the Podkarpackie region of Poland reported total triterpenoid content (UA, OA, BA) ranging from 2892 to 3671 µg/kg dry weight [13], while a similar study conducted on cranberry fruits grown in Latvia reported total triterpene content ranging from 4216 to 6233 µg/g across seven cultivars [17]. A 2022 study of Red Star cultivar *Vaccinium macrocarpon* fruit grown in Lithuania reported 5735.1 µg UA/g dry weight, with the peel as the primary source of UA (6612.8 µg/g), followed by seeds (707.7 µg/g) then pulp (176.2 µg/g) [14]. The reported content of free UA for Red Star is similar to what we measured in most of the cultivars studied herein (Table 2). In a study of phytochemical composition variation among fruits of nine cultivars produced over two seasons in the two different North American growing regions, we did not find a significant effect of cultivar alone on UA and OA content as measured by ^1^H qNMR [15]. On the other hand, the geographical origin had a significant impact on UA and OA content; the average content of UA and OA in fruit of all cultivars was nearly twice as high in fruit from MA than fruit from OR. The same trend is evident among the smaller sampling of fruits in this study (Figure 4). Although studies have reported betulinic acid in cranberries harvested in Poland [13], we detected very little betulinic acid in pomace or in most of the fruit samples in this study compared to the other four triterpenoids. Neutral triterpenoids, including alpha and beta-amyrins [13,18], lupeol, uvaol, and betulin [19], have also been reported at low concentrations in cranberry fruit. Differences in minor triterpenoid constituents were reported for bilberry fruits and leaves (*Vaccinium myrtillus*) grown in Finland compared to plants from Poland, although the same major constituents, UA and OA were present [20]. Triterpenoids are found primarily in the intracuticular wax component of the cuticle, which provides a hydrophobic protective layer covering the aerial parts of plants [21]. As such, environmental conditions can be expected to influence the composition of the wax as well as its protective ability, including permeability and other properties of the cuticle that impact fruit shelf life and storage [21]. The cuticle and its components play an important role in maintaining the health of the plant and the quality of fruits. 

While the majority of studies on cranberry have focused on polyphenols [22], there is reason to believe that the triterpenoids may also contribute to cranberry’s beneficial properties. In addition to earlier studies demonstrating their anti-proliferative effects in cancer cell lines, our recent studies found that cranberry extracts of varying composition reduced the expression of pro-inflammatory cytokines in human THP-1 monocytes and that triterpenoids were among the constituents expected to contribute to these anti-inflammatory properties. In total, eight cranberry extracts were tested for inhibition of LPS-stimulated IL-6 secretion. Two of the most effective extracts in this study (IL-6 inhibition of 34% and 42% at 100 µg/mL extract) were also the highest in UA, OA, and CA content [10]. We previously found that administration of a non-polyphenol extract of cranberry fruit containing primarily triterpenoids and sterols (0.05% *w*/*w* in diet) to AOM-DSS treated mice was able to reduce tumor burden by 40%, with a significant reduction in the expression of pro-inflammatory markers IL-6, IL-1β, and TNF-α in colonic tissue [11]. The observed tumor inhibition and downregulation of inflammatory pathways by UA-rich cranberry extract is consistent with reports of UA’s ability to suppress the proliferation of colon cancer cells through the modulation of multiple pathways including COX2/PGE2 and Akt/ERK [1]. Structure–activity studies have identified UA, OA, and several of their derivatives as effective inhibitors of COX-1, COX-2, 5-LOX, and 15-LOX enzymes [23]. A previous study of cranberry fruit methanolic extract reported the inhibition of the COX-2 enzyme by the extract and attributed this activity to the presence of ursolic acid and its esters, based on activity of an enriched fraction [24]. Interestingly, cis-3-O-p-hydroxycinnamoyl UA isolated from the herbal medicinal plant *Elaeagnus oldhamii* was recently reported to induce apoptosis in oral cancer cells through a p53-mediated mitochondrial pathway [25]. In addition to the many reported effects on tumor cells, OA and UA have demonstrated neuroprotective effects, for example, inhibition of oxidative and inflammatory events in PC12 pheochromocytoma cells, a model of neuroprotection [26]. Several studies in mouse models of neurodegenerative diseases have reported downregulation of the NF-kB pathway in response to long-term treatment with UA, with decreased levels of IL-1β, IL-6, IL-12, and IFN-γ resulting [3], as well as activation of the nuclear factor-erythroid 2-related factor 2 (Nrf2) pathway by UA, which can protect cells from oxidative stress during cerebral ischemia [27,28]. Despite low aqueous solubility, intestinal permeability, and a short plasma half-life, UA, OA, and CA are reportedly able to bioaccumulate in the liver, lung, spleen, colon, and other organs with long-term administration [3,29,30]. 

## 5. Conclusions

The present study indicates that cranberry pomace or presscake are excellent sources of pentacyclic triterpenoid acids, especially UA and OA, suggesting a potential use for an important side stream of fruit processing. Investigation of additional triterpenoid constituents of cranberry pomace is underway. Whole cranberry fruits were also rich in UA and OA, with levels of these compounds varying somewhat between sources. Cis- and trans-hydroxycinnamoyl esters of UA and OA were also detected at high levels, particularly in fruits harvested in MA. Among cranberry products, sweetened dried fruits are a reliable source of these triterpenoids, but cranberry juice beverages provide very low levels of these triterpenoids. The results suggest that cranberry products derived purely from cranberry juice are likely to be much lower in triterpenoids than those prepared from whole fruit, which may help explain the reported variations in content among commercial supplements. Processing methods and fruit sources may affect not only the overall triterpenoid content of cranberry products but also the distribution of esterified vs. free acid forms. As these compounds may be beneficial for colon health and provide protection against other inflammatory conditions, more research is needed to understand how specific processing conditions employed in the manufacture of cranberry-derived products affects their content as well as to understand how environmental factors such as geography and climate influence triterpenoid production in the fruits. 

## Figures and Tables

**Table 1:** Content of major triterpenoids in cranberry products (µg/g product weight ± SD, n = 3).

Sample	Ursolic Acid (UA)	Oleanolic Acid (OA)	Corosolic Acid (CA)	Maslinic Acid (MA)	*cis-* and *trans-*p-Hydroxy-Cinnamoyl UA/OA Esters	Total Triterpenoids
Presscake	6494 ± 29	2133 ± 37	207.5 ± 7.1	55.3 ± 3.3	760 ± 64	9650 ± 58
Pomace	15,500 ± 1700	3990 ± 360	459 ± 21	164 ± 5	615 ± 62	20,738 ± 2089
Fried cranberries	436 ± 7	93.7 ± 1.3	17.6 ± 0.3	3.85 ± 0.03	4.63 ± 0.99	556 ± 7.3
Sweetened dried cranberries	1950 ± 52	452 ± 3.7	45.7 ± 2.0	8.0 ± 0.4	88.4 ± 7.9	2540 ± 65
Whole berry sauce	283.3 ± 4.8	57.1 ± 1.9	7.4 ± 0.1	1.84 ± 0.23	5.7 ± 1.1	355 ± 7.1
Cranberry juice beverage (A)	0.017 ± 0.0013	0.0009 ± 0.0002	ND	ND	ND	0.018 ± 0.001
Cranberry juice beverage (B)	0.190 ± 0.007	0.054 ± 0.001	0.0081 ± 0.0005	0.0031 ± 0.00013	ND	0.26 ± 0.008
Cranberry juice beverage (C)	0.013 ± 0.0037	0.003 + 0.0009	0.0009 ± 0.0003	0.0007 ± 0.0001	ND	0.018 + 0.005
Cranberry juice beverage (D)	0.191 ± 0.014	0.052 ± 0.0009	0.0216 ± 0.0017	0.0070 ± 0.00006	ND	0.27 ± 0.017

ND = not detected.

**Table 2:** Content of major triterpenoids in cranberry materials and fruits (µg/g dry weight ± SD, n = 3).

Sample	Ursolic Acid (UA)	Oleanolic Acid (OA)	Corosolic Acid (CA)	Maslinic Acid (MA)	*cis-* and *trans-*p-Hydroxy-Cinnamoyl UA/OA Esters	Total Triterpenoids
Presscake	27,770 ± 120	9120 ± 160	887 ± 30	237 ± 14	3236 ± 270	41,250 ± 248
Pomace	47,520 ± 5100	12,220 ± 1100	1960 ± 91	502 ± 15	1885 ± 190	64,090 ± 6420
Fried cranberries	437 ± 7	93.9 ± 1.3	17.6 ± 0.3	3.86 ± 0.03	4.6 ± 1.0	557 ± 7.3
Whole berry sauce	610 ± 10	123 ± 4.0	15.96 ± 0.2	3.97 ± 0.50	12.3 ± 2.4	765 ± 15
Dried cranberries	2040 ± 54	474 ± 4	46.9 ± 2.1	8.41 ± 0.40	92.8 ± 8.3	2665 ± 68
Yellow Bell fruit	8360 ± 320	2040 ± 110	518 ± 25	95 ± 3.2	807.8 ± 42.5	11,703 ± 612
Early Black fruit (MA, 2017)	7423 ± 183	2650 ± 63	925 ± 19	225 ± 8	5844 ± 51	17,070 ± 320
Stevens fruit (MA, 2016)	5236 ± 90	1858 ± 70	107 ± 12	20.0 ± 3.5	2875 ± 114	10,095 ± 289
Stevens fruit (MA, 2017)	5909 ± 129	2132 ± 53	123 ± 10	48.4 ± 2.3	4325 ± 174	12,537 ± 368
Welker fruit (MA, 2016)	5435 ± 83	1511 ± 52	48.8 ± 9.7	6.5 ± 2	1910 ± 37	8911 ± 188
Welker fruit (MA, 2017)	6996 ± 46	2640 ± 87	265 ± 14	72.5 ± 1	1235 ± 93	11,209 ± 241
Welker fruit (OR, 2016)	5511 ± 94	1764 ± 39	117 ± 29	105 ± 4	58 ± 14	7555 ± 180
Welker fruit (OR, 2017)	5244 ± 98	1632 ± 13	108 ± 2.6	48.4 ± 1.4	160 ± 10	7192 ± 125
GH-1 fruit (MA, 2016)	5961 ± 170	2306 ± 46	100 ± 1.9	19.9 ± 0.7	7055 ± 290	15,441 ± 493
GH-1 fruit (MA, 2017)	6733 ± 325	2470 ± 88	69.2 ± 10	22.7 ± 2.9	5671 ± 434	14,970 ± 860
GH-1 fruit (OR, 2016)	4971 ± 100	1444 ± 18	45.9 ± 1.1	12.8 ± 0.4	68 ± 7	6542 ± 119
GH-1 fruit (OR, 2017)	5174 ± 87	1510 ± 2	51.8 ± 1.9	13.9 ± 1.9	211 ± 32	6961 ± 125

## Data Availability

The original contributions presented in the study are included in the article/Supplementary Materials, further inquiries can be directed to the corresponding author.

## Supplementary Materials

The following supporting information can be downloaded at https://www.mdpi.com/article/10.3390/foods13193136/s1, Figure S1: Representative TIC and EIC chromatograms at [M-H]^−^ = 455.4; Figure S2A: Extracted mass spectrum for OA; Figure S2B: Extracted mass spectrum for UA; Figure S3: Representative TIC and EIC chromatograms at [M-H]^−^ = 471.4, Figure S4A: Extracted mass spectrum for MA; Figure S4B: Extracted mass spectrum for CA; Figure S5: Extracted mass spectrum for cis-hydroxycinnamoyl ester of OA; Figure S6: Extracted mass spectrum for cis-hydroxycinnamoyl ester of UA; Figure S7: Extracted mass spectrum for trans-hydroxycinnamoyl ester of OA; Figure S8: Extracted mass spectrum for trans-hydroxycinnamoyl ester of UA.
