# Supplementary figures and images for: Pentacyclic Triterpenoid Content in Cranberry Raw Materials and Products
Source: Foods. 2024 Sep 30;13(19):3136. doi: 10.3390/foods13193136 (PMC11475460; doi:10.3390/foods13193136)

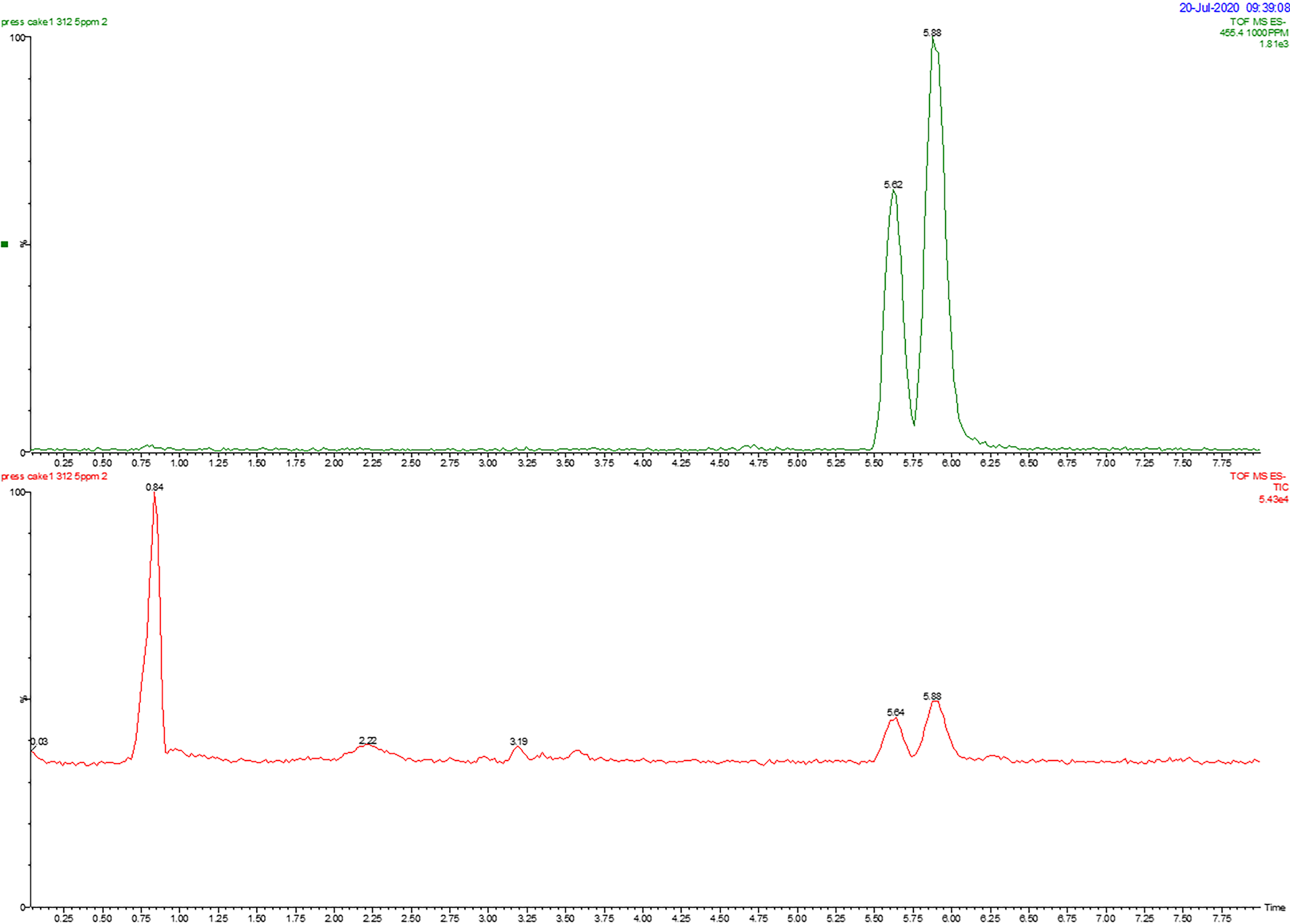

Supplement: Supplementary file 1 [file foods-13-03136-s001.zip › Figure S1.tif]

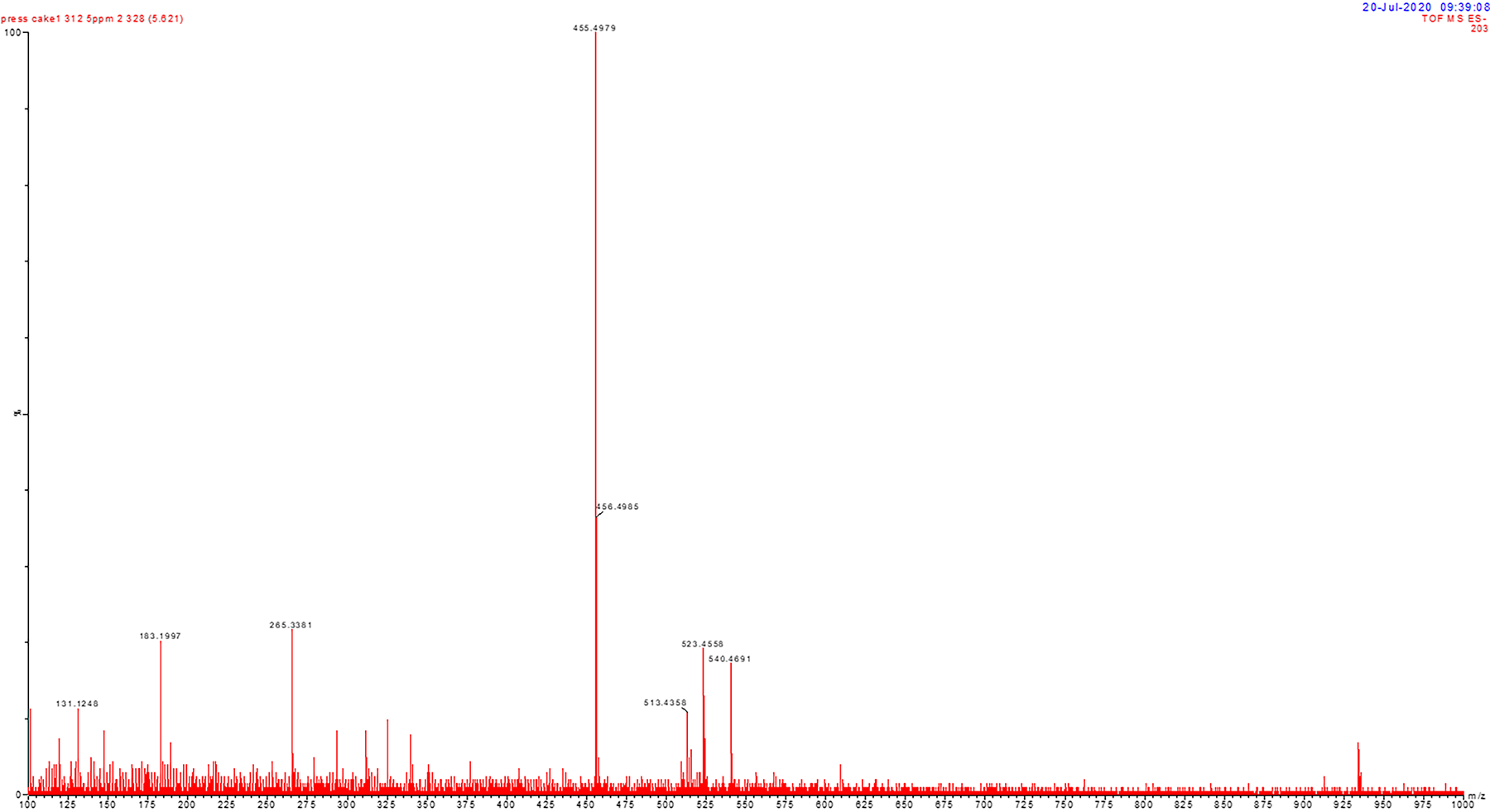

Supplement: Supplementary file 1 [file foods-13-03136-s001.zip › FIgure S2A.tif]

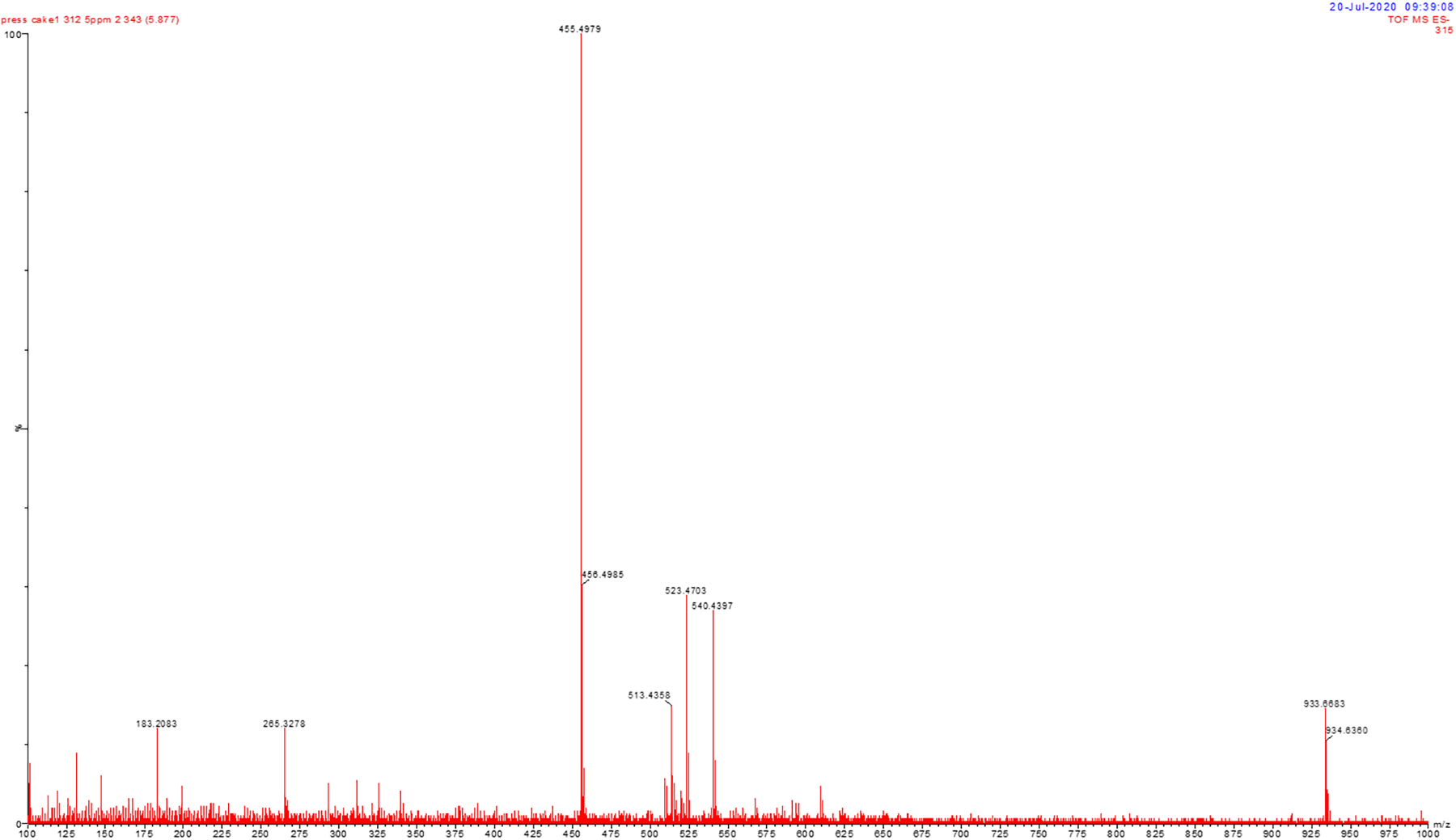

Supplement: Supplementary file 1 [file foods-13-03136-s001.zip › Figure S2B.tif]

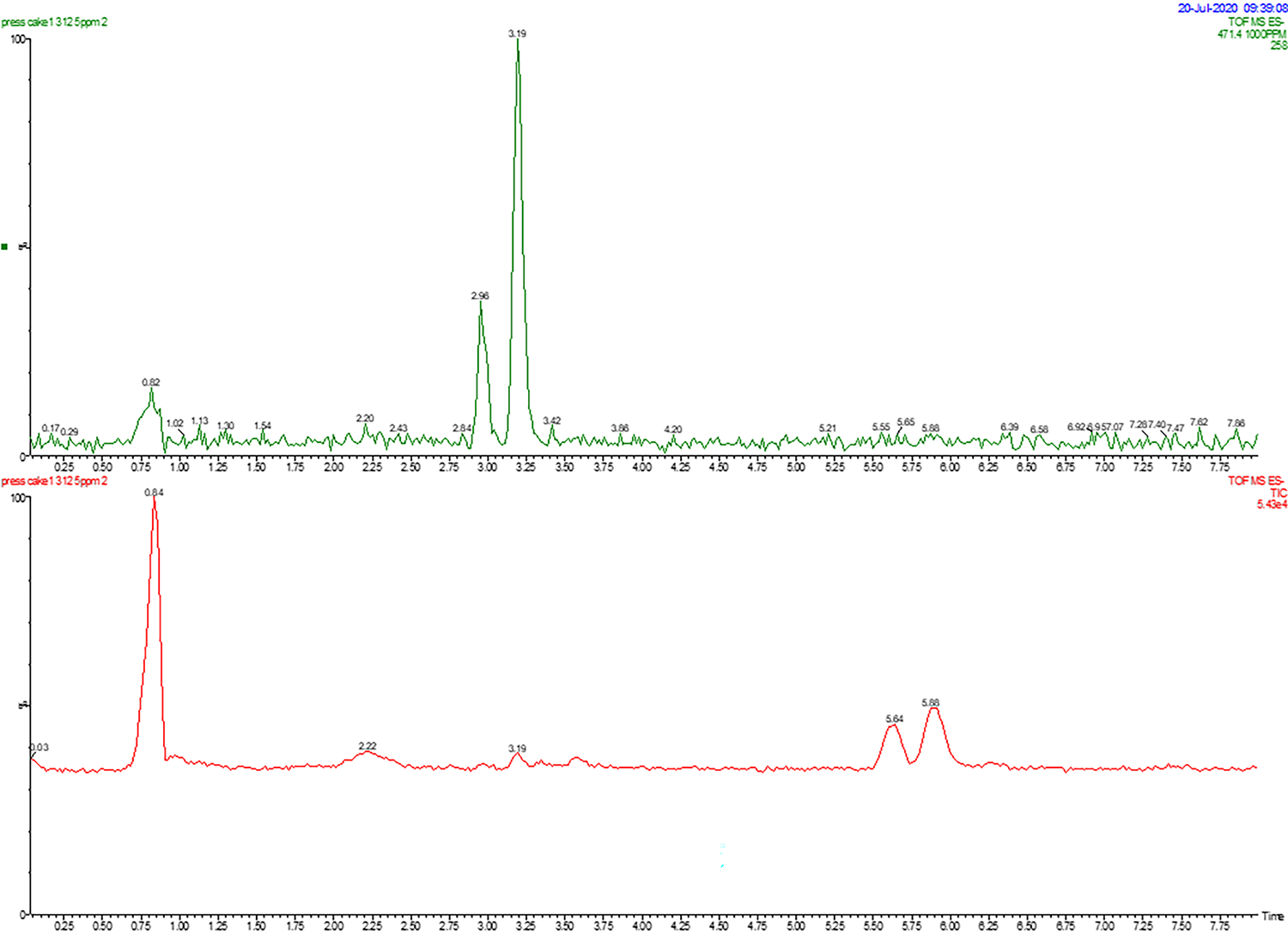

Supplement: Supplementary file 1 [file foods-13-03136-s001.zip › Figure S3.tif]

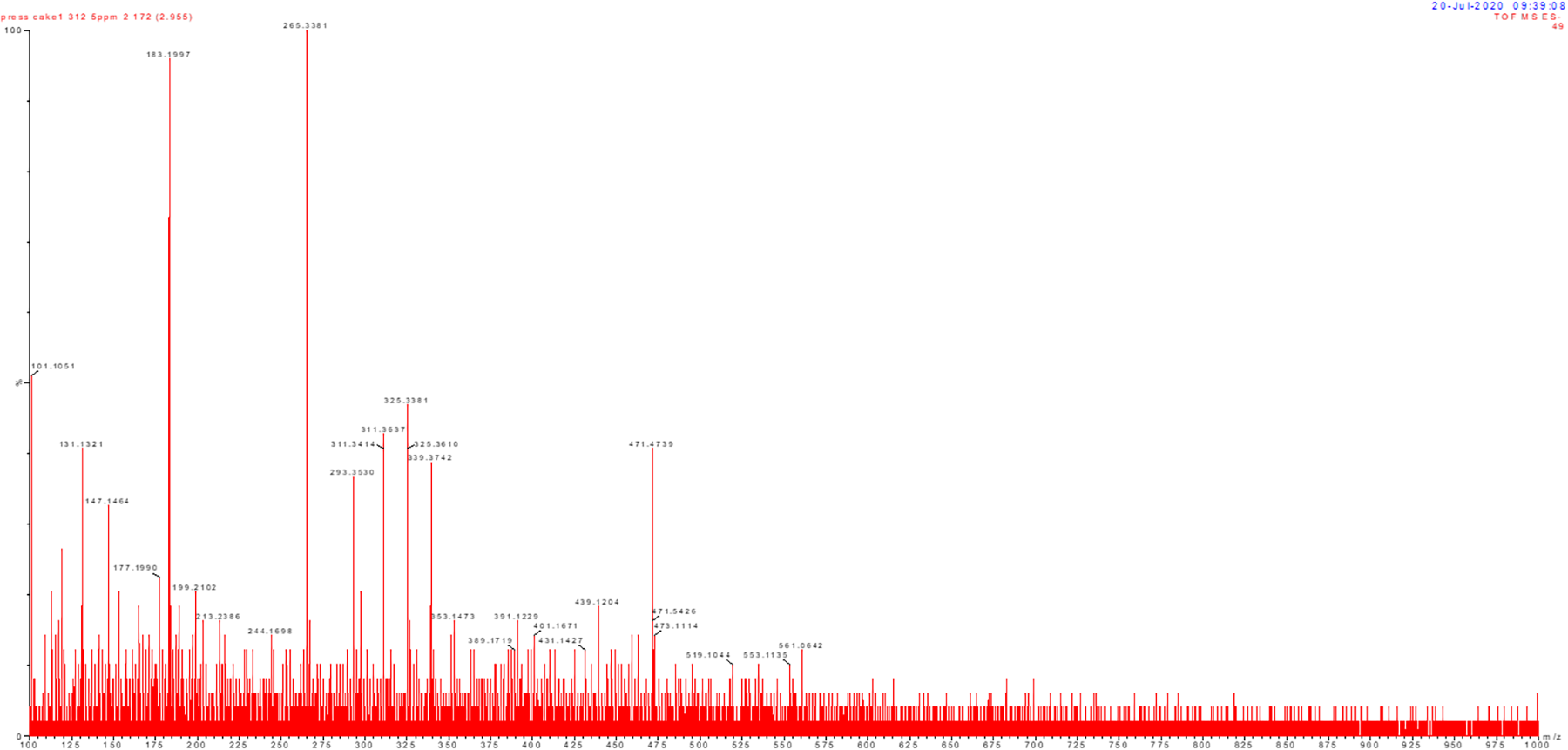

Supplement: Supplementary file 1 [file foods-13-03136-s001.zip › Figure S4A.tif]

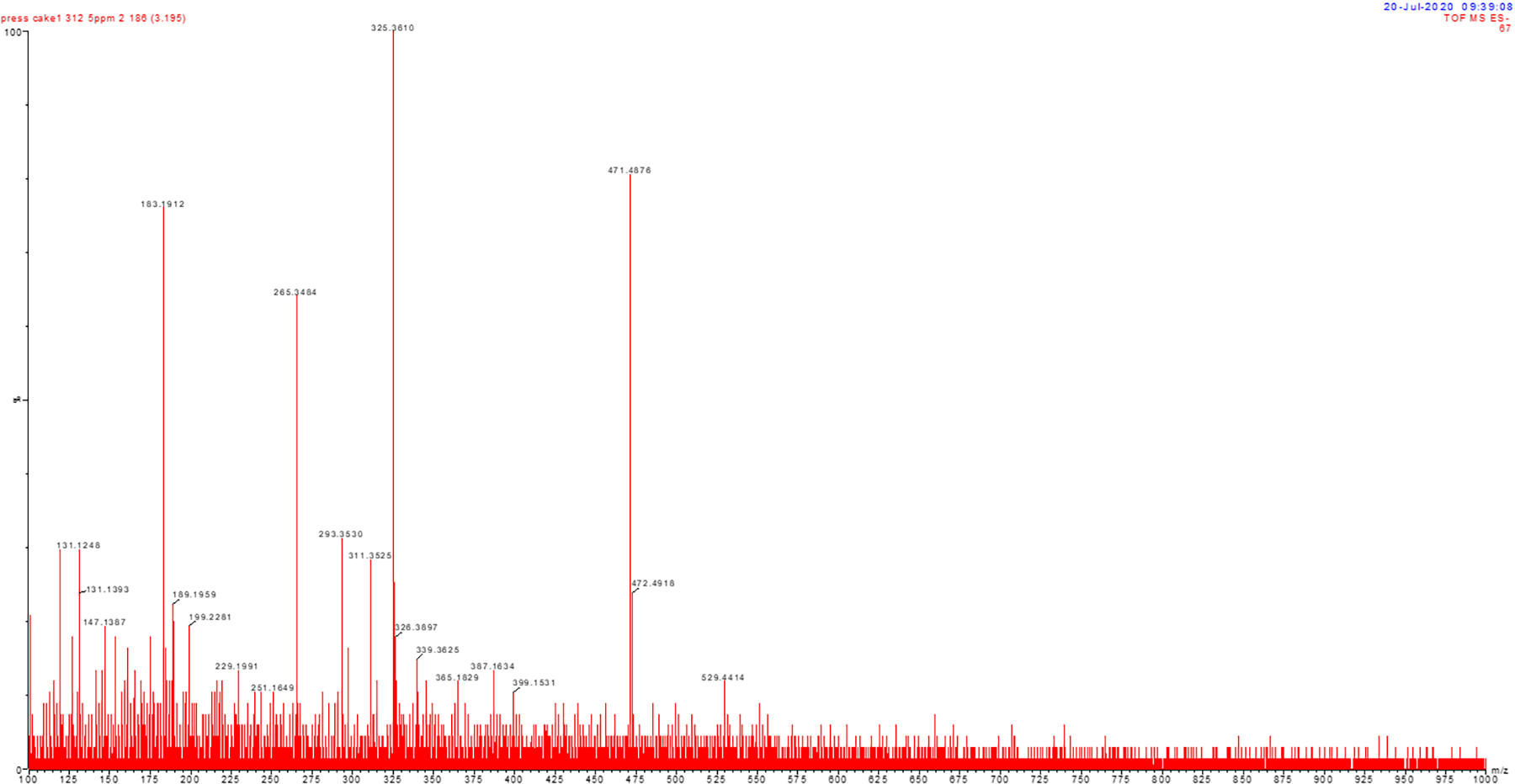

Supplement: Supplementary file 1 [file foods-13-03136-s001.zip › Figure S4B.tif]

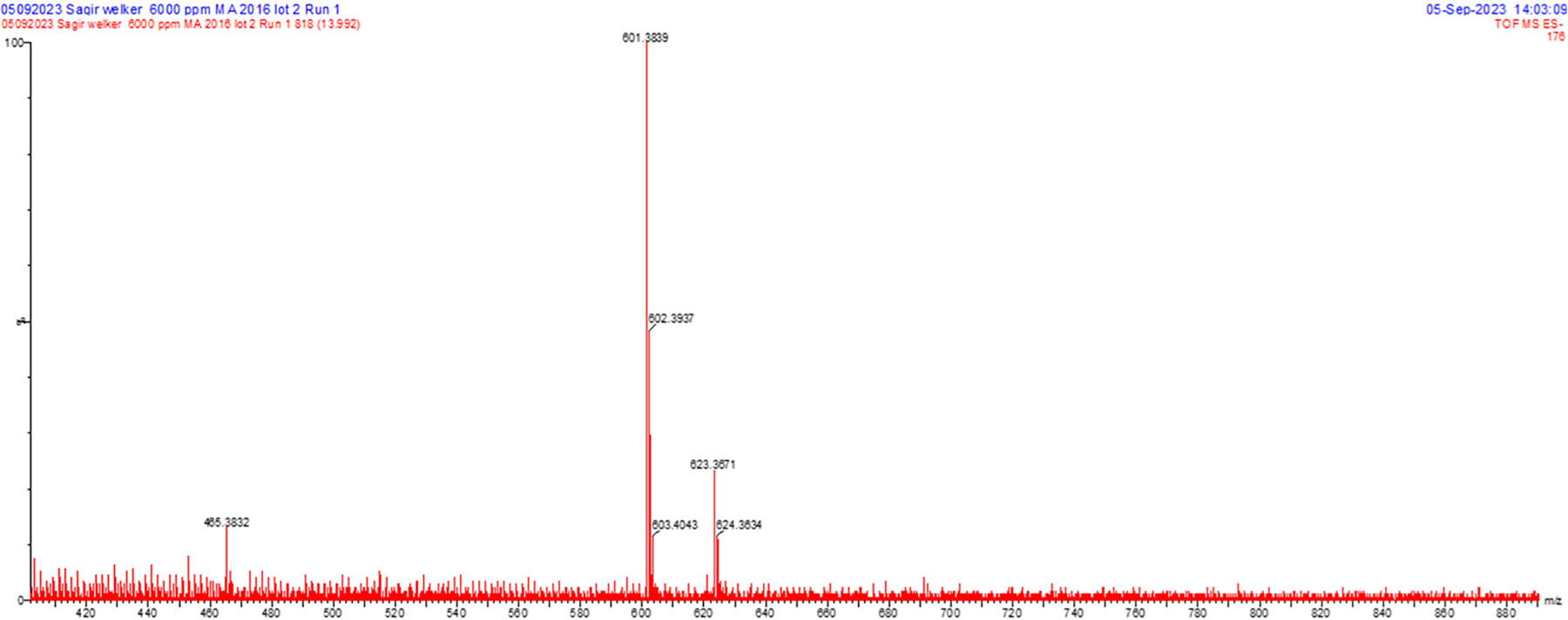

Supplement: Supplementary file 1 [file foods-13-03136-s001.zip › Figure S5.tif]

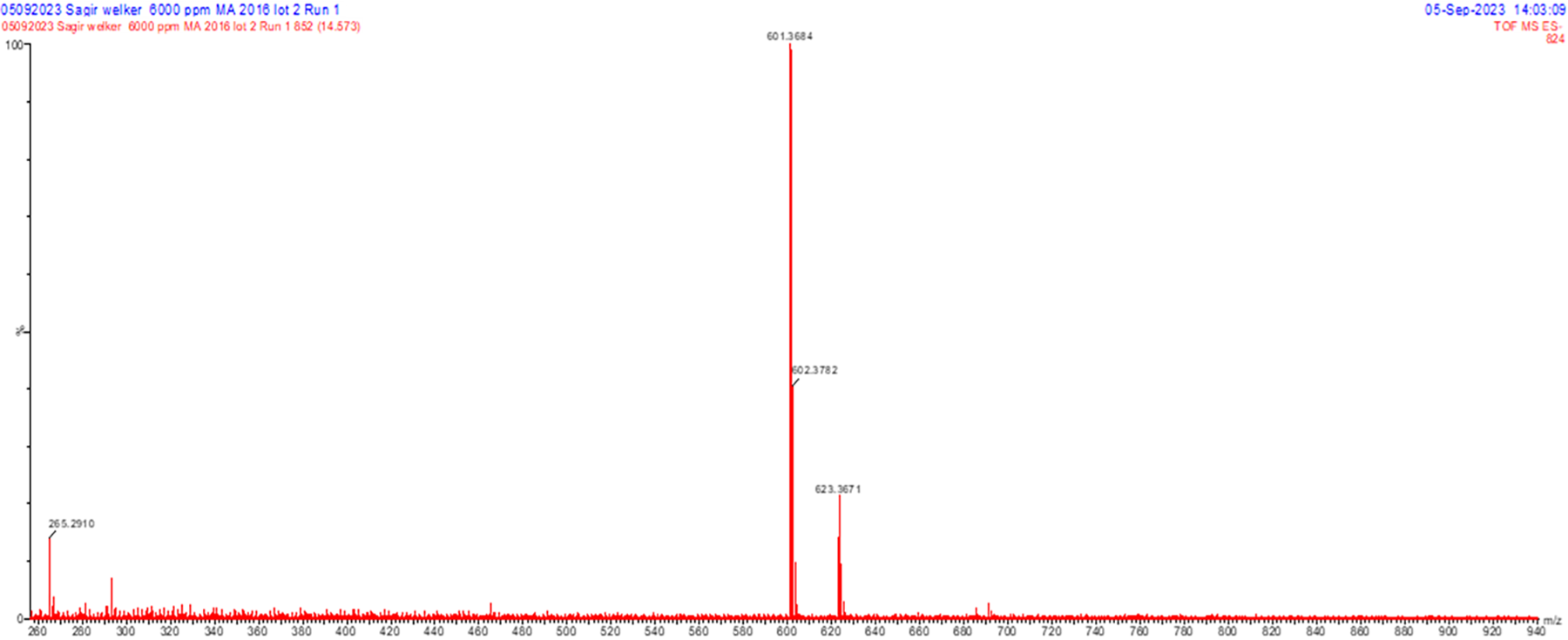

Supplement: Supplementary file 1 [file foods-13-03136-s001.zip › Figure S6.tif]

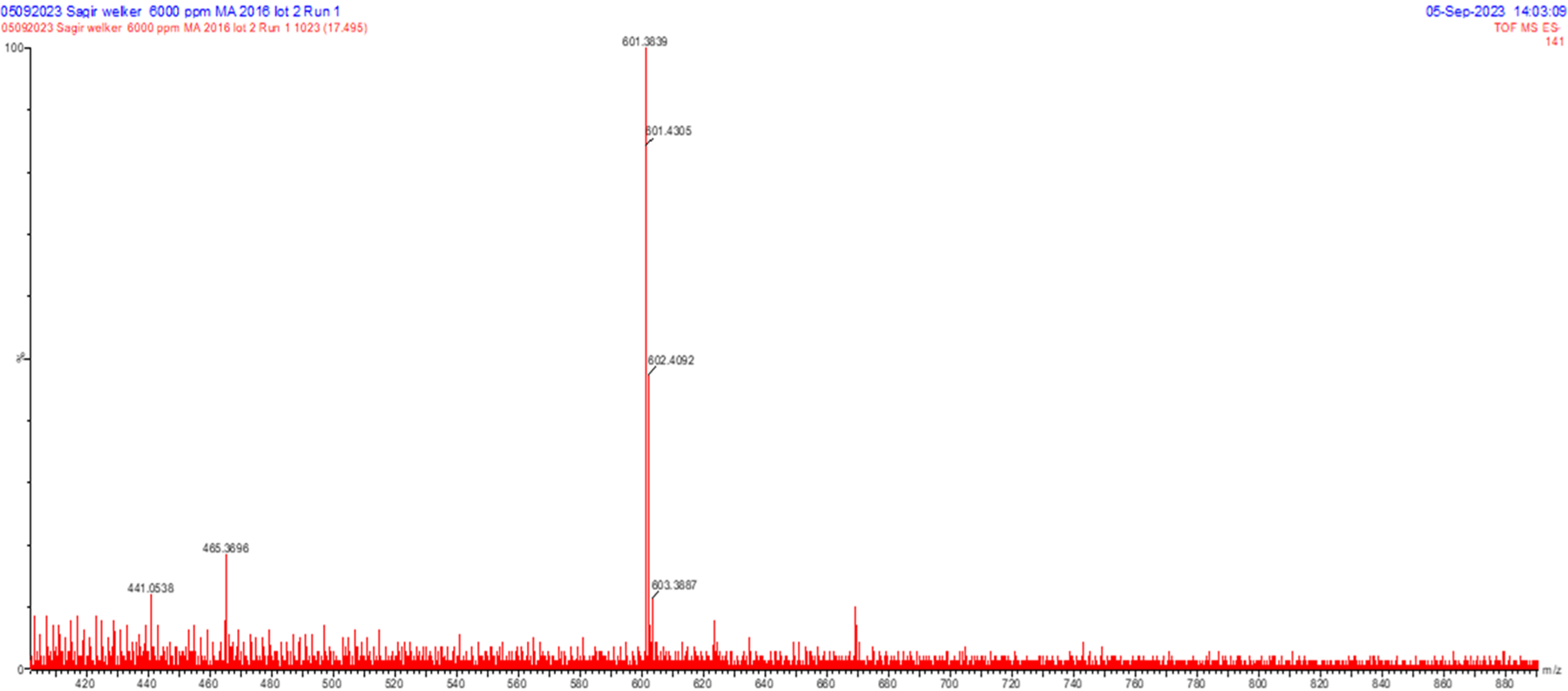

Supplement: Supplementary file 1 [file foods-13-03136-s001.zip › Figure S7.tif]

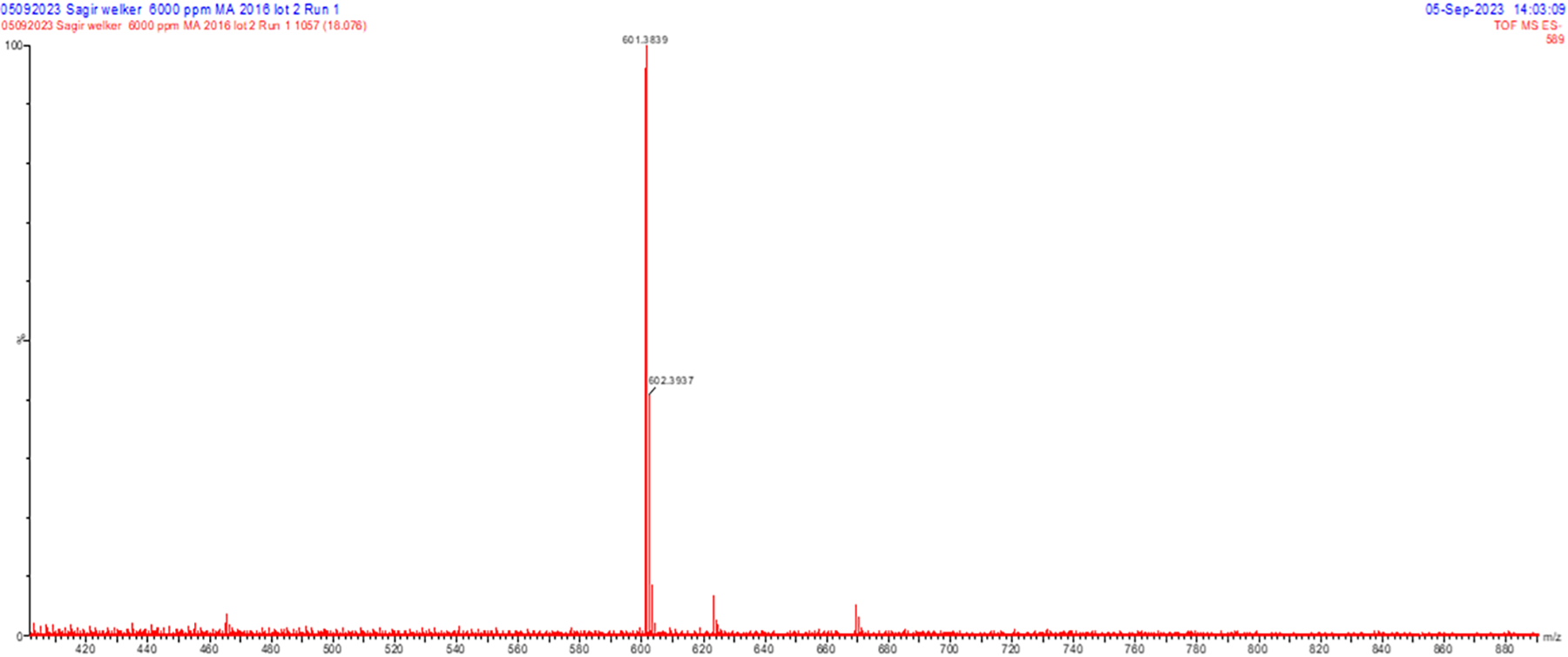

Supplement: Supplementary file 1 [file foods-13-03136-s001.zip › Figure S8.tif]
